# Acute oral toxicity of pesticide combination (acephate 50% and imidacloprid 1.8% as active ingredients) in Sprague-Dawley rats

**DOI:** 10.14202/vetworld.2018.1291-1297

**Published:** 2018-09-18

**Authors:** Rajendra Palkhade, Suresh Yadav, SukhDev Mishra, Jaseer Muhamed

**Affiliations:** 1 Laboratory Animal Facility, ICMR-National Institute of Occupational Health, Ahmedabad, Gujarat, India; 2 Toxicology Division, ICMR-National Institute of Occupational Health, Ahmedabad, Gujarat, India; 3 Biostatistics and Data Management Division, ICMR-National Institute of Occupational Health, Ahmedabad, Gujarat, India; 4 Poison Information Center, ICMR-National Institute of Occupational, Ahmedabad, Gujarat, India

**Keywords:** acephate 50%, acute oral toxicity, histopathology, imidacloprid 1.8%

## Abstract

**Aim:**

The aim of this study was to assess the acute toxic interaction and lethal dose (LD_50_) of pesticide combination product (acephate 50% and imidacloprid 1.8% as active ingredients) available in the market in Sprague-Dawley female rats by oral route.

**Materials and Methods:**

A total of 10 Sprague-Dawley female rats were divided into two groups, comprising five rats in each dose group. Both groups were identified as control and test groups, respectively. Control group received sterile water as vehicle and test group received pesticide combination (acephate 50% and imidacloprid 1.8% as active ingredients) at a dose of 0 and 2000 mg/kg body weight. As per the Organization for Economic Cooperation and Development Guideline 420, initially one animal each from both the control and test groups were dosed with 0 and 2000 mg/kg, respectively, as sighting study. Based on the results of sighting study, additionally, four animals each from both groups were dosed with the same dose to make a total of five animals in each group. Dose volume was constant as 10 mL/kg. All animals were observed daily twice for clinical signs and mortality. Body weight was recorded on day 0 and weekly thereafter during 14 days’ observation period; last body weight (fasted) was recorded on day 15. All the rats of both the groups were humanely sacrificed on day 15 for gross pathology, collection of organs for histopathology, organ weighing, and morphometry. Organ weights were taken as absolute values, and relative organ weights to last fasted body weights were calculated.

**Results:**

Pesticide combination (acephate 50% and imidacloprid 1.8% as active ingredients) treated rats showed cholinergic signs with one mortality in the test group. No significant difference was observed in body weight, relative organ weights, and organ morphometry between pesticide combination exposed and non-exposed groups. Gross pathology of the treated rats was also comparable with respect to control group. Histopathological changes in the liver, kidneys, heart, lung, adrenaline, spleen, and ovaries of test group rats were found to be comparable with control group rats.

**Conclusion:**

The present study demonstrated the LD_50_ of one of the combination products available in the market having acephate 50% and imidacloprid 1.8% as active ingredients in Sprague-Dawley female rats which is >2000 mg/kg body weight. Furthermore, gross, histopathology and histoarchitectural alterations of all the vital organs of the test group were comparable to the control.

## Introduction

Pesticides have numerous beneficial effects such as crop protection, preservation of food and materials, and prevention of vector-borne diseases. Average application rate of pesticide arable land (per hectare) was highest average values 6.5-60 kg/ha, occurred in Asia and some countries of South America as computed by the Food and Agriculture Organization [1]. Indiscriminate and unregulated the use of pesticide with mere traditional knowledge without use of personal protective equipment is a common practice among farmers in developing countries. Also, mixing different pesticides without technical knowledge for more production and profit lead to occupational hazards [2]. Besides the occupational hazards of acute exposure to pesticide, self-poisoning with agriculture pesticides is one of the major contributors to the global burden of suicide [3]. Acute pesticide poisoning is now an important cause of morbidity and mortality worldwide, especially in the developing countries [4]. There are strong grounds for concern that official estimates of the incidence of suicide in India may be underestimated [5]. The current paradigm for the risk assessment of chemical substance focuses on the effects of individual substances for determining the doses of toxicological concern to inform appropriately for regulatory reasons. Concerns about potential toxicity from multiple chemical (combination pesticide) exposure are rising [6]. Farmers are at risk of exposure of combination pesticides through dermal contact and inhalation while mixing, loading, spraying, and cleaning up of spraying equipment [7].

The use of cocktail pesticides or practice of mixing different classes of pesticides with the suggestion of shopkeeper or other farmers to achieve better efficacy and more crop production is being a common practice among Indian farmers. One of the most important reason for using a combination or mixture of pesticide is to delay resistance built up for any class of insecticide on plants along with its synergistic effects against the pest [8]. As demand increases for the combination pesticides by the end users, the pesticide manufacturing companies are also started to prepare the combination pesticides. To date, about 36 combination pesticide products have been registered under restricted use category at the Central Insecticide Board and Registration Committee (CIBRC), India [9]. To ensure the safety of combination pesticides, toxicological hazard evaluation of combination pesticides is necessary than evaluations of individual chemical components separately. Unhealthy practices with pesticides in agriculture sector lead to contamination of soil and food crops with pesticides and can result in pesticide contamination of food [10]. In view of environmental contamination by chemicals, their entry into air, water and food chain, WHO has made a framework for the risk assessment of combined exposures to multiple chemicals [11].

Combination pesticides are widely used in India, and various registered products are being marketed which constitute acephate 50% and imidacloprid 1.8% as active ingredients, under restricted use category and marketed by different trade names, especially for use in cotton crop. As per the WHO classification, acephate is classified in Class II group [12]. The acephate is an organophosphate group of insecticides which was introduced as nerve poison or chemical warfare agents during the World War II [13]. Methamidophos, the primary metabolite of acephate, a potent inhibitor of acetylcholinesterase enzyme results in accumulation of acetyl choline in nerve tissue causing hypercholinergic symptoms in mammals. Acephate exhibits excellent insecticidal activity but low mammalian toxicity (oral LD_50_ for rats 2851 mg kg body weight) [14]. Imidacloprid belongs to neonicotinoids group of pesticides. The group strongly suspected to be involved in the collapsing of bee colonies. It is registered as a pesticide first in the US by the United States Environmental Protection Agency and is one of the best-selling insecticide [15]. Imidacloprid works through activation of nicotinic acetylcholinesterase receptors, ultimately causing paralysis and death. It is moderately toxic to mammals including humans compared to many older synthetic pesticides. However, it is highly toxic to other non-target and insect species also. Selective toxicity of imidacloprid in insects is due to differences in the binding affinity or potency at the nicotinic acetylcholine receptors [16]. However, data are lacking on toxicity evaluation for acephate and imidacloprid pesticides in combination. Hence, there is an urgent need to evaluate the acute toxicity of this combination pesticide (acephate 50% and imidacloprid 1.8% as active ingredients).

The aim of the present study was to determine the acute toxic effects of pesticide combination of acephate 50% and imidacloprid 1.8% as active ingredients in rats. Acute toxicity, prominent clinical signs, body weight, relative organ weight, morphometry of organs, gross, and histopathological changes in vital organs were evaluated following acute oral dosing of the combination pesticide as per the Organization for Economic Cooperation and Development (OECD) Guidelines 420 [17].

## Materials and Methods

### Ethical approval

Animal ethical clearance was obtained from the Institutional Animal Ethics Committee of the ICMR-National Institute of Occupational Health, Ahmedabad, vide no (IAEC/NIOH/2014-15/03).

### Chemicals

Pesticide combination product available in the market (as it is) of having acephate 50% and imidacloprid 1.8% as active ingredients, alkyl naphthalene sulfonate as surfactant, and precipitate silica as other ingredients were purchased from the registered pesticide dealer and used in the experiment.

### Experimental animals

Ten Sprague-Dawley female rats (8-9 week old) were procured from Zydus Research Center, Ahmedabad (registered with CPCSEA, New Delhi, India). The animals were acclimatized for 7 days before initiation of the study in the institution’s laboratory animal facility. All rats were housed in polypropylene cages with rice husk as bedding material, fed *ad libitum* with standard pellet feed (Pranav Agro Industries, Pune, India), and have *ad libitum* access of sterilized and filtered water. The room was maintained under a 12/12 h light-dark cycle, an ambient temperature at 19-25°C, and relative humidity at 45% (±15). A total of 10 female rats were randomized and divided into two groups on day 0, comprising five females in each group for two dose levels, i.e., 0 and 2000 mg/kg body weight in control and test groups, respectively. Female animals used in the study were nulliparous and non-pregnant.

### Doses of the compounds

The aqueous solution of the test substance was prepared by dissolving it in distilled water. Dose was given keeping the volume constant to 10 mL/kg body weight as per the OECD guideline for acute dose toxicity testing 420 [17]. Based on the literature survey of acute toxicity of individual pesticide, the dose selected was 2000 mg/kg body weight in comparison with the control group of 0 mg/kg body weight. The dose was calculated on the basis of body weight recorded on the day “0” at the time of randomization and grouping. Since the pesticide combination used is 50% acephate and 1.8% imidacloprid as active ingredients, the effective dose used in the study was equivalent to 1000 mg/kg acephate and 36 mg/kg imidacloprid.

### Sighting study

As per the OECD Guidelines, following the period of overnight fasting, single animal from each group was administered with the respective dose. Both animals survived.

### Main study

Based on the results of sighting study, remaining four female rats from each group were dosed, i.e., 0 and 2000 mg/kg body weight (“known as a Limit test”).

During and after the experiments, the following parameters were studied: The animals of the test and control groups were observed at 1/2, 1, 2, 4, 6, 12, and 24 h after the treatment and thereafter twice daily up to 14 days for morbidity and mortality patterns. The health conditions of the animals were recorded by a qualified veterinary practitioner experienced in the field of experimental toxicology.

### Body weight gain effects

Body weights of all animals were recorded on day 0, thereafter on weekly interval up to 14 days’ observation period, and finally, on day 15 (fasted), before necropsy.

### Gross necropsy

At the time of termination of the study on day 15, all animals were euthanized by CO_2_ asphyxiation. Gross necropsy was carried out after dissection and soaking the moisture with the help of tissue paper and examined for gross pathological changes and recorded individually.

### Relative organ weight and morphometric measure- ment

The wet weights of internal organs (heart, lung, liver, kidney, adrenaline, spleen, and ovaries) were recorded as absolute values and calculated relative to last fasted body weight.

The values of morphometric measurement, i.e., length, breadth, and thickness of various organs (heart, liver, spleen, and kidney) were measured with the help of Vernier caliper to know any adverse effect of pesticide combination on the organ morphometry.

### Histopathology

After gross examination, the vital organs (liver, lung, thymus, kidneys, spleen, ovary, uterus, heart, intestine, stomach, and brain) of rats were processed for possible histopathological examination with Hematoxylin and Eosin stain (H and E stain). The tissues were fixed in 10% formalin for 3 days. The fixative was removed by washing through running tap water for overnight. After dehydration through a graded series of alcohol, the tissues were cleared in xylene and embedded in paraffin wax. Sections were cut of 5 µ thickness and stained with H and E and observed under a light microscope for histopathological changes.

### Statistical analysis

The data generated on various parameters are reported as mean and standard error. To evaluate the statistical significance of various continuous variables, t-test was applied between the control and treated groups. In case of the non-normality of data, Wilcoxon rank-sum test was applied, and all tests were carried out at 5% level of significance.

## Results

### Animals

All animals survived from both the groups until day 15 except one animal from the test group which was found dead within 24 h of dosing.

### Toxicological signs

#### Sighting study

Both control and test group animals survived after a dose of 0 and 2000 mg/kg body weight, respectively. The test group animals showed toxicological signs typical of cholinergic syndromes including dullness, tremors, muscular weakness, ruffled fur, lethargy, lacrimation, exophthalmia, and gasping (Table-1). After 1 h, the toxicological sign was boosted with piloerection, and as time passes, the signs also increased with abdominal breathing. However, on 2^nd^ day (after 24 h of dosing), the toxicological signs were reduced, and only piloerection was observed and continued until day 7.

**Table-1:** Toxicological effect of the acute exposure of pesticide combination which constitutes acephate 50% and imidacloprid 1.8% on single animal in sighting study.

Animal ID	Sighting study dose	Clinical signs	
01	2000 mg/kg body weight by oral gavage	30 min	No signs
		1 h	Piloerection
		2 h	Tremors and piloerection
		4 h	Tremors and piloerection
		6 h	Abdominal breathing, tremors, piloerection, and muscular weakness
		12 h	Tremors, piloerection, and lacrimation
		24 h	Tremors and piloerection
		Up to 7 days	Animal recovered from toxicological signs except piloerection which was continued until 7 days
		Up to 14 days	Animal recovered, whereas piloerection was continued until 14 days

#### Main study

As one animal of the sighting study survived at the dose of 2000 mg/kg body weight, remaining four animals were dosed at the dose of 2000 mg/kg with concurrent control group to have a total of five animals. The test group animals revealed the same cholinergic toxicological signs with typical cholinergic syndrome including dullness, whole-body tremors, muscular weakness, ruffled fur, lethargy, lacrimation, and exophthalmia, and after 1 h, it was supplemented with piloerection. The severity was somewhat higher in animal ID #4 of test group and the same animal was found dead within 24 h of treatment. Rest of the animals (total 4) survived and recovered well from toxicological signs after day 7 except piloerection which was continued until day 14 (Table-1). No abnormality was detected in any of the animals of control group which received distilled water only.

### Body weight

Body weight of animals of both the groups was recorded on day “0,” thereafter on weekly intervals up to 14 days’ observation period, and finally, on day 15 as fasted body weights (Table-2). The body weight at weekly interval of the treated group rats did not reveal any significant difference when compared with the control group.

**Table-2:** Effect of acute oral administration of pesticide combination (acephate 50% and imidacloprid 1.8%) on body weight (g) gain in female rats.

Group	Weekly body weight of animals		

	Day 0	Day 7	Day 14
Control	261.8±3.12	281.4±5.22	284.8±5.67
Test	260.6±2.94	275.75±55.66	288.5±58.43
p-value	0.841 (NS)	0.222 (NS)	0.843 (NS)

Values are given as Mean±SE, SE=Standard error, NS=Not significant

### Median lethal dose (LD_50_)

The LD_50_ of the combination pesticide (acephate 50% and imidacloprid 1.8% as active ingredients) in rat is found to be >2000 mg/kg body weight and it passed the limit test. During the course of experiment, only one animal was found dead at a dose of 2000 mg/kg body weight, and hence, the said test substance is categorized into “5” as per globally harmonized system (GHS) for the classification of chemicals when the study was conducted as per the modified OECD Guidelines 420 [17].

### Relative organ weight and morphometric measure- ment

No significant difference was observed in relative organ weight of test group rats when compared with control group rats (Table-3). The values of morphometric measurement of the various organs of test group rats also not differed significantly when compared to the control group (Table-4).

**Table-3:** Effect of acute oral administration of pesticide combination (acephate 50% and imidacloprid 1.8%) on relative organ weight (g/100 g of body weight) of organs in female rats.

Organs	Control (0 mg/kg)	Treatment (2000 mg/kg)	p-value
Heart	0.33±0.025	0.35±0.031	0.899 (NS)
Lung	0.56±0.014	0.64±0.066	0.861 (NS)
Liver	2.85±0.174	2.88±0.015	0.905 (NS)
Spleen	0.20±0.019	0.19±0.010	0.908 (NS)
Kidneys	0.61±0.032	0.58±0.030	0.907 (NS)
Ovaries	0.05±0.005	0.04±0.001	0.413 (NS)
Adrenal glands	0.02±0.001	0.02±0.001	0.286 (NS)

Values are given as Mean±SE, SE=Standard error, NS=Not significant

**Table-4:** Effect of acute oral administration of pesticide combination acephate 50% and imidacloprid 1.8% on various organs morphometry data in cm of female rats.

Organ name	Control (0 mg/kg body weight)			Treatment (2000 mg/kg body weight)		
	
	Length	Breadth	Thickness	Length	Breadth	Thickness
Heart^#^	1.5±0.037	1.0±0.017	0.7±0.007	1.2±0.184	1.0±0.802	0.6±0.164
Liver^#^	4.8±0.066	3.4±0.025	0.5±0.009	3.7±0.535	2.7±0.381	0.39±0.056
Spleen^#^	3.6±0.180	0.6±0.004	0.3±0.009	3.1±0.434	0.4±0.061	0.2±0.038
Kidney^#^	0.8±0.009	1.7±0.038	0.6±0.005	0.6±0.088	1.2±0.182	0.5±0.075

Values are given as Mean±SE,

# No statistical significance was observed between groups.

### Gross pathology of vital organs

At the time of sacrifice of animals, the gross pathology of all vital organs was carried out and no major changes were observed in test group rats when compared with the control group.

### Histopathology

Microscopically, the organs (liver, lung, thymus, kidneys, spleen, ovary, uterus, heart, intestine, stomach, and brain) of the test group were comparable to the control group. No abnormalities were observed in both control and test group animals.

## Discussion

Regulatory test for pesticide safety for the registration purpose is currently only done on active ingredient(s), assuming that other ingredients have no effects, for example, ignoring adjuvant effect(s) [18], and to avoid this in the present study, we have tested whole combination pesticide product (acephate 50% and imidacloprid 1.8% as active ingredients) as it is available in the market. Pesticides combination (two or more) may have additive, synergistic, or antagonistic effect. Rats exposed to the combination of acephate 50% and imidacloprid 1.8% exhibited cholinergic signs within the 1^st^ week of dose. This cholinergic symptom may be because of the presence of acephate as one of the active ingredients in the combination pesticide. This consists of a sequence of neurological signs that appeared within 24-36 h, after the acute cholinergic crises [19]. In the present study, it was observed that the toxicological signs such as lacrimation, whole-body tremors, muscular weakness, and ruffled fur in rat after 2 h of dosing; similar symptoms were also reported by Gupta and Moretto [20] who reported that the LD_50_ of acephate orally as 1400 mg/kg body weight in rats and toxic signs disappeared at day 8 in surviving rats. They have further mentioned that no gross pathological alterations were seen in rats at necropsy. Kumar [14] also observed cholinergic signs in an acute toxicity study of acephate individually in rats. The toxic effects observed in the present study within a short duration after exposure are attributed to chemobiokinetic properties of acephate as mentioned by Kumar [14]. It is reported that acephate has been rapidly absorbed, metabolized, and excreted from the body [21]. Similarly, imidacloprid also almost completely absorbed from the gastrointestinal tract and eliminated via urine and feces within 48 h [22]. In the present study, this may be the reason that other animals recovered within 7 days of dosing except one animal which found dead within 24 h of dosing. Another possible reason might be individual animal’s physiology failed to excrete acephate rapidly comparative to other (four) rats which survived until day 14. The mechanism of rapid absorption and excretion was also supported by Kumar [14], who mentioned that the presence of an efficient detoxification mechanism in rats plays an important role. The LD_50_ of technical grade acephate through oral route is 1400 (670-2800) and 1000 (490-2100) mg/kg body for male and female rats, respectively [23]. Doss and Mohiyuddin [24] mentioned that clinical signs of acephate toxicity appeared within 15 min of dosing and recovery was observed within 8-24 h. To some extent, our results follow the similar trend and required more than 24 h duration to recover from the cholinergic signs. The LD_50_ of pesticide combination (acephate 50% and imidacloprid 1.8% as active ingredients) is >2000 mg/kg body weight in Sprague-Dawley female rats as per the OECD Guideline 420 (fixed dose method) [17]; the pesticide combination (acephate 50% and imidacloprid 1.8% as active ingredients) was classified into category-five as per the GHS. Crisp and Look [25] reported LD_50_ of acephate as 866-945 mg/kg, Worthing and Walker [26] reported oral LD_50_ of acephate in rats as 850 mg/kg, and Kumar [14] mentioned the median LD of acephate as 2851 mg/kg body weight when tested individually in Wistar rats. Death of one animal of five indicates that the acephate presence may be responsible for lethality at the dose rate of 2000 mg/kg body weight. The difference of lethality in the present study may be due to the use of different strain of the experimental animals, and the earlier researcher used the acephate and imidacloprid individually, whereas in this study, we used acephate 50% along with imidacloprid 1.8% in combination with other inert ingredients. Acute oral toxicity of acephate is medium (500-5000 mg/kg body weight) to high (50-500 mg/kg body weight) as per Briggs [27]. As per the United States Environment Protection Agency classification [28], the imidacloprid is moderately toxic to rat which supports our claims regarding moderately toxic classification. Sheets [29], evaluated that imidacloprid toxicity individually also showed similar results to what we observed in the present study that in Sprague-Dawley rat; no death and no gross lesion were recorded on necropsy; all rats gained body weight, and LD_50_ of imidacloprid is >4820 mg/kg body weight. In the acute toxicity study, they have found LD_50_ as 2591 and 1858 mg/kg body weight in Sprague-Dawley male and female rats, respectively. Furthermore, they have also observed whole-body tremors, labored breathing, stained fur salivation, and lacrimation and resolved on day 14, which is also similar to the finding of toxicological signs and symptoms.

Bagri *et al*. [30] found non-toxic effects on hematological parameters, body weight, relative organs weight, and growth profile at 110 mg/kg body maximum tolerated dose of imidacloprid in mice. It is also reported that imidacloprid is moderately toxic at the dose rate of 425-475 mg/kg body weight in rat, mice of different strains and supports our findings regarding moderately toxic compound [31]. The present study on histopathological alteration in all organs was comparable to the control. Similarly, Nellore *et al*. [32] did not found any histopathological changes in different regions of rat brain at the dose rate of 80 mg/kg of imidacloprid in a single and double dose. Most studies suggest that a combination of pesticides always has an additive effect, and when two or more pesticides are used together, their toxicity is always more than the toxicity of single pesticide [33]. Moderately toxic effects observed in the present study may be due to the fact that, acephate do not show synergism at dietary exposure [34]. Groten *et al*. [35] suggested that a mixture should be evaluated by testing each compound separately and thereafter different combinations of the compounds. Testing of combination pesticide as per Groten *et al*. [35] would be possible to identify the compound(s) responsible for possible interactions. In the present study, the two pesticides (acephate and imidacloprid) were not tested individually or in different combinations. It cannot, therefore, be concluded whether the toxic effects observed initially (1^st^ week) was caused by one or two of the pesticides present in the combination pesticide. However, the present study data demonstrated similar to one of the principals of risk assessment of combination compound that exposure to a combination of compounds does not cause effects stronger than the ones of their most active component, and the provided components are present at low concentration levels.

## Conclusion

Based on the results of this study, the acute oral LD_50_ of acephate 50% and imidacloprid 1.8% in female rats is found to be >2000 mg/kg body weight, when tested as per the OECD Guidelines 420, and the test substance is classified in “category-5” as per the GHS.

The trend of toxicological signs we have observed in acute toxicity test was probably due to the toxic effect of acephate and imidacloprid combination. We also want to conclude with a positive note that the LD_50_ estimation with this method will also save a number of animal’s life in comparison with the traditional method of the estimation of LD_50_. Furthermore, research studies targeting long-term exposure of acephate 50% and imidacloprid 1.8% are warranted, and the data of the present study will be important one while conducting long-term (subacute and subchronic) toxicity study.

## Authors’ Contributions

RP designed and conducted the experiments. RP, SY, and SM analyzed the results. RP and JM drafted and reviewed the manuscript. All authors read and approved the final manuscript.
